# A Practical Treatise on Midwifery

**Published:** 1844-06

**Authors:** 


Art. IV.—*A Practical Treatise on Midwifery.* By M. Chailly, M.D., and Ex-Chief of the Obstetric Clinique of the Faculty of Paris, Prof. of Midwifery, Member of the Society of Medical Emulation, &c., &c., &c. Illustrated with 216 wood-cuts. A work adopted by the Royal Council of Public Instruction. Translated from the French and Edited by Gunning S. Bedford, M.D., Professor of Midwifery and the Diseases of Women and Children, in the University of New York. New York: Harper & Brothers. 1844. 12mo. pp. 530.

The full title page enumerates the claims of this work to the confidence of the profession: it was adopted by the Council of Public Instruction of France, and was esteemed of so much practical value by an American teacher of midwifery, that he undertook the labor of presenting it to his countrymen in an English dress. Professor Bedford further states that the volume embodies the instructions and experience of the eminent Paul Dubois, which, he justly supposes, will secure for it a favorable reception on this side of the Atlantic. In some respects, the publishers have done justice, in the mechanical execution, to the high scientific character of the work, but the wood-cuts have been a good deal criticised, and we must say with propriety. We wonder that pains were not taken to render the volume more unexceptionable in this particular. As it is, however, the student will derive a great advantage from the illustrations, which are given on every few pages. Combining, as the work does, all that is new in obstetric science, and abounding in that practical information which the young accoucheur wants at the bed side, it may be recommended as one of the most compendious treatises on the subject before the profession. Y.

